# P-534. Similar Efficacy, Safety and CD4 T-cell Increase up to Week 96 Observed with Fostemsavir (FTR)-Based Regimens in the BRIGHTE Study and Dolutegravir (DTG)-Based Regimens in the VIKING-3 Study in Individuals with Multidrug-Resistant (MDR) HIV-1

**DOI:** 10.1093/ofid/ofae631.733

**Published:** 2025-01-29

**Authors:** Antonella Castagna, Natalia Gregori, Iacopo Marcon, Fangfang Du, Bo Li, Marcia Wang, Alftan Dyson, Clifford B Jones, Manyu Prakash, Andrew Clark

**Affiliations:** IRCCS San Raffaele Hospital and Vita-Salute San Raffaele University, Milano, Lombardia, Italy; ViiV Healthcare, Verona, Veneto, Italy; ViiV Healthcare, Verona, Veneto, Italy; Temple University, Chesterbrook, Pennsylvania; GSK, Collegeville, Pennsylvania; GlaxoSmithKline, Upper Providence, Pennsylvania; ViiV Healthcare, Verona, Veneto, Italy; ViiV Healthcare, Brentford, UK, London, England, United Kingdom; ViiV Healthcare, Brentford, UK, London, England, United Kingdom; ViiV Healthcare, Brentford, UK, London, England, United Kingdom

## Abstract

**Background:**

Constructing suppressive regimens in individuals with MDR HIV-1 can be challenging. We assessed populations with limited ART options in the BRIGHTE study using FTR-based regimens and the VIKING-3 study using DTG-based regimens.

**Methods:**

BRIGHTE was a phase 3 study (N=371; Randomized Cohort [RC], n=272; Non-randomized Cohort, n=99) in adults failing current ART (HIV-1 RNA > 400 c/mL) with ≤ 2 fully active approved ARVs. Participants (pts) with 1 or 2 active ARVs entered the RC and received open-label FTR + optimized background therapy (OBT) after an 8-day blinded placebo-controlled period. In the RC, the most common agent in initial OBT was DTG (84%), with 64% taking it twice daily (BID). VIKING-3 (n=183) was a single-arm open-label phase 3 study in adults with INI-resistant virus receiving DTG 50 mg BID + failing regimen (without raltegravir or elvitegravir) through Day 7, then the regimen was optimized with ≥ 1 fully active ARV and DTG continued. Virologic and immunologic response was analyzed by baseline (BL) demographics and characteristics.

**Results:**

BRIGHTE pts were male (n=290, 78%), median age 48 years and White (n=259, 70%). Observed antiviral response in the RC was 81% (< 40 c/mL, n=128) and 88% (< 400 c/mL, n=140). Mean CD4 increase from BL was 204.7 cells/mm^3^, with BL mean CD4 count 152.5 cells/mm^3^. VIKING-3 pts were male (n=141, 77%), median age 48 years and 71% (n=130) White. Antiviral response observed in VIKING-3 was 84% (< 50 c/mL, n=101) and 93% (< 400 c/mL, n=111). Mean CD4 increase was 192 cells/mm3, with BL mean CD4 count 202 cells/mm^3^. Safety across study populations was as follows: BRIGHTE (RC), n=92 (34%) serious adverse events (AEs), n=57 (21%) drug-related AEs and n=7 (3%) AEs leading to withdrawal; VIKING-3, n=60 (33%) serious AEs, n=52 (28%) drug-related AEs and n=8 (4%) AEs leading to withdrawal.

**Conclusion:**

Despite limited ARV options in individuals with MDR HIV-1, data demonstrate that over 96 weeks both DTG (BID) and FTR-based regimens provide robust viral suppression and CD4 T-cell improvement. Although differences in BL characteristics exist, immunosuppression was more profound in BRIGHTE pts. FTR and DTG were commonly used together in BRIGHTE, engaging different mechanisms of action. Data provide further support for effective ARVs for individuals living with MDR HIV-1.

**Disclosures:**

**Antonella Castagna, MD**, Bristol-Myers Squibb: Advisor/Consultant|Gilead Sciences, Inc.: Advisor/Consultant|Gilead Sciences, Inc.: Grant/Research Support|Gilead Sciences, Inc.: Honoraria|Janssen: Advisor/Consultant|Janssen: Grant/Research Support|Janssen: Honoraria|Merck Sharp & Dohme: Advisor/Consultant|Merck Sharp & Dohme: Grant/Research Support|Merck Sharp & Dohme: Honoraria|ViiV Healthcare: Advisor/Consultant|ViiV Healthcare: Grant/Research Support|ViiV Healthcare: Honoraria **Natalia Gregori, MD**, GSK: Stocks/Bonds (Public Company)|ViiV Healthcare: employee **Iacopo Marcon, PhD**, GSK: Stocks/Bonds (Public Company)|ViiV Healthcare: employee **Fangfang Du, MS in Statistics**, GSK: Employee|GSK: Stocks/Bonds (Public Company) **Bo Li, PhD**, GSK: Employee **Marcia Wang, PhD**, GSK: Employee|GSK: Stocks/Bonds (Public Company) **Alftan Dyson, PharmD**, GSK: Stocks/Bonds (Public Company)|ViiV Healthcare: Employee **Clifford B. Jones, BSc MSc MB ChB**, GSK: Stocks/Bonds (Public Company)|ViiV Healthcare: Employee **Manyu Prakash, PhD**, GSK: Stocks/Bonds (Public Company)|ViiV Healthcare: employee **Andrew Clark, MD**, GSK: Stocks/Bonds (Public Company)|ViiV Healthcare: employee

